# Precocious initiation of spermatogenesis in a 19-month-old boy with Hurler syndrome

**DOI:** 10.1186/2051-4190-24-8

**Published:** 2014-05-01

**Authors:** Jean-Pierre Milazzo, Amandine Bironneau, Jean-Pierre Vannier, Agnes Liard-Zmuda, Bertrand Macé, Rives Nathalie

**Affiliations:** Reproductive Biology Laboratory - CECOS, Rouen University Hospital, Rouen, F-76031 France; EA 4308 “Gametogenesis and Gamete Quality”, IRIBHN, University of Rouen, Rouen, F-76000 France; Service d’Immuno-Hémato-Oncologie pédiatrique, Rouen University Hospital, Rouen, F-76031 France; Département de chirurgie infantile, Rouen University Hospital, Rouen, F-76031 France; Reproductive Biology Laboratory - CECOS, Rouen University Hospital, 1 rue de Germont, Rouen, F-76031 France

**Keywords:** Androgen receptor, Anti-Müllerian hormone, Hurler syndrome, Spermatogenesis, Testicular tissue, Récepteur aux androgènes, Hormone anti-müllérienne, Syndrome de Hurler, Spermatogenèse, Tissu testiculaire

## Abstract

Mucopolysaccharidosis type IH (MPS IH) is a rare autosomal recessive lysosomal storage disorder. Haematopoietic stem cell transplantation (HSCT) has been proposed for the treatment of MPS IH patients and offers the possibility to grow into their adulthood. Precocious puberty has been described in few MPS patients. We report, to the best of our knowledge and for the first time, the initiation of the first waves of spermatogenesis fortuitously observed in seminiferous tubules of a pre-pubertal 19-month-old boy, affected by MPS IH and who did not present any clinical signs of precocious puberty. This patient benefited from testicular tissue cryopreservation before HSCT. Seminiferous tubule size, germ cell differentiation and Sertoli cell expression of androgen receptor and anti-müllerian hormone corresponded to the pattern observed in a pubertal boy. The Hurler syndrome may be responsible for the precocious initiation of spermatogenesis. A specific follow-up during childhood may be useful to confirm if such abnormal testis development is common in young boys with MPS IH and if it may lead to precocious onset of puberty in survivors despite HSCT. Furthermore, we have observed that Sertoli cell maturation (up-regulation of AR expression, down-regulation of AMH expression) occurred before the clinical signs of puberty and before the increase of testosterone plasmatic level.

## Résumé

La Mucopolysaccharidose de type IH (MPS IH) est une maladie rare lysosomale de transmission récessive autosomique. L’allogreffe de cellules souches hématopoïétiques (CSH) a été proposée pour le traitement des patients atteints de MPS IH et offre la possibilité d’une survie de ces patients jusqu’à l’âge adulte. La puberté précoce a été décrite chez quelques patients MPS. Nous rapportons, à notre connaissance et pour la première fois, l’initiation de la première vague de spermatogenèse observée fortuitement dans les tubules séminifères d’un garçon de 19 mois, atteint de MPS IH et sans signes cliniques de puberté précoce. Ce patient a bénéficié d’une cryoconservation de tissu testiculaire avant la greffe de CHS. Le diamètre des tubes séminifères, la différenciation des cellules germinales et l’expression du récepteur aux androgènes et de l’hormone antimüllérienne dans les cellules de Sertoli présentent les caractéristiques retrouvées dans le testicule d’un garçon pubère. Le syndrome de Hurler peut être responsable de l’initiation précoce de la spermatogenèse. Un suivi spécifique pendant l’enfance peut être utile pour confirmer si cette maturation prématurée du testicule est fréquente chez les jeunes garçons atteints de MPS IH et si elle peut conduire à une puberté précoce chez les patients malgré la greffe de CSH. En outre, notre observation démontre que la maturation des cellules de Sertoli se produit avant les signes cliniques de puberté et avant l’augmentation de la concentration plasmatique de testostérone.

## Background

Hurler syndrome (HS), or mucopolysaccharidosis type IH (MPS IH), is a rare autosomal recessive lysosomal storage disorder, due to α-L-iduronidase activity deficiency, enzyme required for the breakdown of the glycosaminoglycans (GAG) heparan and dermatan sulfates [1]. MPS IH clinical phenotype is due to an excessive accumulation of GAG in lysosomes of affected organs. Clinical signs usually appear before the age of 2 years and the median survival is 6.8 years without any treatment [2]. Enzyme replacement therapy and haematopoietic stem cell transplantation (HSCT) are the treatment proposed in MPS IH and offer the chance for children with MPS IH to grow into their adulthood. Consequently, long-term outcomes and complications have become clinically relevant [3–6].

Central precocious puberty (CPP) [7] has been exceptionally described in patients with type IH or IIIA MPS [8–10] and central nervous system organic lesions are frequently observed within this context [11].

We report, for the first time and to the best of our knowledge, the fortuitous observation of precocious initiation of the first waves of spermatogenesis in a pre-pubertal 19-month-old boy with MPS IH who underwent testicular tissue cryobanking before HSCT, in order to preserve his future fertility. Spermatogenesis was compared with data obtained in four boys, aged between 1 and 17 years, who cryopreserved testicular tissue within the context of non-malignant disease.

## Case presentation

### Patient

Our HS patient was the first child of healthy non-consanguineous parents. MPS IH was diagnosed at 15 months, due to cornea clouding, inguinal hernia, respiratory disorders, hepatosplenomegalia and dorsal kyphosis. Brain magnetic resonance imaging revealed mild ventricular enlargement and cystic areas of corpus callosum splenium region. Deficiency of α-L-iduronidase activity with two missense mutations of the gene (W402X and M504R) confirmed the diagnosis. The patient received enzyme replacement therapy and HSCT was performed at 20 months. Testicular tissue cryopreservation was proposed before HSCT and the patient did not exhibit any clinical signs of puberty (Tanner 1 stage) [12].

Plasma Luteinising Hormone (LH) and Follicle-Stimulating Hormone (FSH) were measured by chemiluminescent immunoassay (Immulite 2500, Siemes Healthcare Diagnostics), testosterone by radioimmunoassay (in duplicate) (Immunotech Beckman-Coulter, Marseille, France), inhibin B by ELISA (Ge II ELISA Beckman Coulter) as well as Anti-Müllerian Hormone (AMH) by immunoassay (EIA Immunotech Beckman-Coulter). The lower limit of sensitivity for LH and FSH was 0.1 IU/l. The detection limit was 0.1 ng/ml for testosterone, 2.6 pg/ml for inhibin B and 1 ng/ml for AMH.

At the time of the biopsy (19 months), the plasmatic levels of inhibin B and AMH were increased. After HSCT, basal plasmatic FSH level was higher than the 95 percentile for pre-pubertal normal boys (Tanner stage I). However, LH, testosterone, inhibin B and AMH levels were normal (Table 1).

**Table 1:** Hormonal findings before (at the time of the biopsy) and after haematopoietic stem cell transplantation (HSCT) of the Hurler syndrome patient * [32]

	Age (years)	FSH basal (mUI/ml)	LH basal (mUI/ml)	Testosterone basal (ng/ml)	AMH (ng/ml)	Inhibin B (pg/ml)
Before HSCT	1.8	0.47	0.27	0.1	121.2	239
After HSCT	2.2	2.6	0.3	<0.1	57.8	112
*Normal values*	*1.5*	*0.1-2.5*	*0.2-2.95*	*<0.3*	*51.38-88.34*	<182*

### Testicular tissue samples

Four boys, aged between 1 and 17 years, who cryobanked testicular tissue due to non-malignant disease, were considered as controls (C) for the histological analysis of testicular tissue. These controls (C1, C2, C3 and C4) were not previously exposed to gonadotoxic treatment before testicular tissue banking and they presented thrombopaenia (C1), drepanocytosis (C2), aplastic anaemia (C3) and torsion of the left testis (C4) respectively. Parents or legal guardians signed an informed consent for testicular tissue cryopreservation, analysis of the tissue and publication of data collection. Our study respects the current bioethics French law for the preservation of germinal tissue.

### Testicular tissue preparation

A bilateral testicular biopsy was performed under general anaesthesia and was cut into 10 mg fragments. The testicular fragments were placed into cryovials containing the cryoprotective medium and were frozen using a controlled slow freezing protocol without seeding in a programmable freezer (Freezal®, Air Liquide, Paris, France), before transfer into liquid nitrogen [13, 14]. One fragment of each biopsy was fixed in Bouin’s solution and was used for histological evaluation. Ten sections (4 μm) of each testis were cut at 20 μm intervals and stained with haematoxylin-eosin-saffron (HES) or processed for immunochemistry. Observations were performed using a conventional light microscope (Diaplan, Leica Microsystem, Solms, Germany) with serial digital images recorded at × 1000 magnification. The results were expressed as the mean of the data obtained from the two testes for all the evaluated parameters.

### Histological analysis of testicular tissue

The mean diameter and area of 30 cross-sectioned seminiferous tubules were determined using a digital image analysis system (LAS 2.8.1, Leica, Solms, Germany). A tubule was defined as cross-sectioned when the ratio between the longest diameter and the diameter perpendicular to the longest one was evaluated between 1 and 1.5.

Five-micrometer sections of paraffin-embedded tissue were boiled for 40 min in 0.01M-citrate buffer, pH 6 (S2031, Dako, Trappes, France). Slides were incubated for 5 min in HP block solution (S2023, Dako, France) and overnight at 4°C with (i) 1:100 of rabbit anti- Androgen Receptor (AR) primary antibody (sc-816, Santa Cruz, CA, USA), or (ii) 1:50 of goat anti-AMH primary antibody (6886, Santa Cruz, CA, USA), or (iii) 1:50 of rabbit anti 3β-hydroxysteroid dehydrogenase (3β-HSD) (E-0353, Dako, Les Ulis, France) or 25 min at room temperature (RT) with 1:300 of mouse anti-Vimentin primary antibody (M0725, Dako, Trappes, France). Bound antibody was detected using an avidin biotin complex and 3.3′-diaminobenzidine (K5001, Dako, Trappes, France). Negative controls were obtained by omitting primary antibody. Stained cells were counterstained with Haematoxylin (Dako, France) and analysed under a light microscope (DM 4000 B, LEICA, Solms, Germany) at ×400 magnification.

AMH labelling was scored according to the staining intensity and the proportion of intra-tubular stained area. AR labelling was scored for myoid, Leydig and Sertoli cells according to the intensity and the proportion of stained cells. Sertoli cell maturity was qualitatively assessed as follows: (i) following AMH labelling, the Sertoli cells were considered to be infantile for complete intratubular area staining, intermediate for partial staining and in post-pubertal state for negative staining, (ii) following AR labelling, the Sertoli cells were considered to be in an infantile state if no staining was observed, in an intermediate state if a cytoplasmic staining was detected and in post-pubertal state if intense Sertoli cell nuclear staining was observed, (iii) following vimentine labelling, Sertoli cell morphology was considered to be infantile if the nuclei were round to ovoid with one or two small nucleoli and post-pubertal if the nuclei were most often triangle-shaped, with a large, centrally located nucleolus [15, 16].

## Results

**Figure 1:**
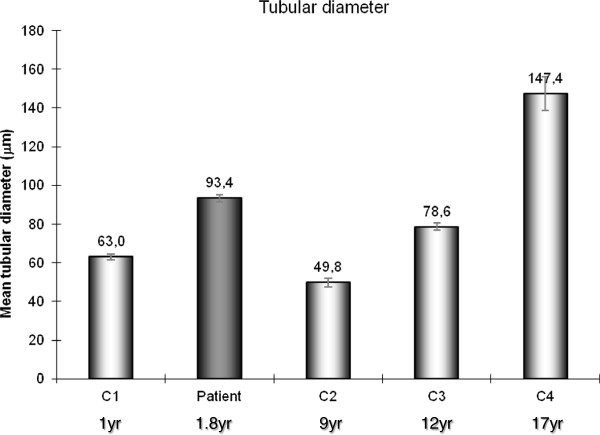
**Mean seminiferous cord size of the Hurler syndrome patient and controls according to the age at the time of the testicular biopsy.** Data are presented as mean±standard deviation (SD).

**Figure 2:**
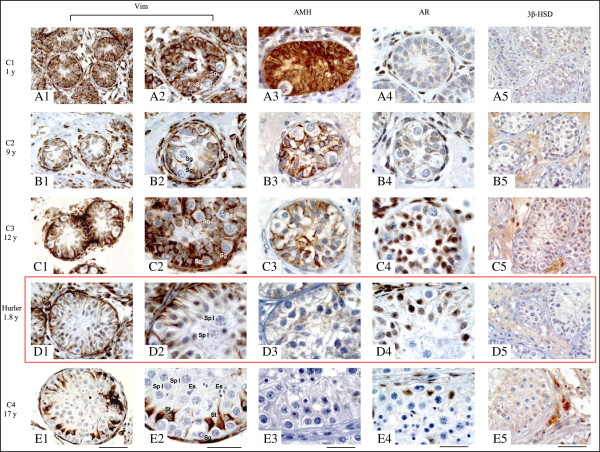
**Sertoli cell immuno-labelling of vimentine [1 and 2], anti-Müllerian hormone [3], androgen receptor [4] and Leydig cell immuno-labelling of 3β HSD: 3β-hydroxysteroid dehydrogenase [5] in seminiferous tubules of the Hurler syndrome patient [D] and 1.8 [C1 – A], 9 [C2 – B], 12 [C3 – C] and 17 [C4 – E] -year-old controls respectively.** Sertoli cells with positive expression of (i) cytoplasmic vimentine or AMH as well as (ii) nucleic AR, are brown stained. For Hurler patient and controls, AR was always expressed in Leydig and myoid cells. AR could be detected in Sertoli cells only at the onset of puberty [C4 and E4] and in our patient [D4]. AMH was strongly expressed for the youngest control [A3], weakly expressed for our Hurler patient [D3] and undetectable for the oldest control [E3]. 3β-HSD was weakly expressed in Leydig cells of the Hurler patient [F3], moderately expressed at the onset of puberty [B5 and C5] and highly expressed in the oldest control [E5]. Scale bar: (1, 3, 4) 50 μm, (2) 30 μm. *Footnotes:* AMH: Anti-Müllerian Hormone - AR: Androgen Receptor - 3β-HSD: 3β-hydroxysteroid dehydrogenase - C: Control - Es: Elongated spermatid - Sg: Spermatogonia - Sp I: Spermatocyte I - St: Sertoli cell - Vim: Vimentine - y: years.

The tubule diameter increased with age in controls and was comprised between the values observed in the 12 and 17-year-old controls, in our HS patient (Figures 1 and 2A1, B1, C1, D1 and E1).

**Figure 3:**
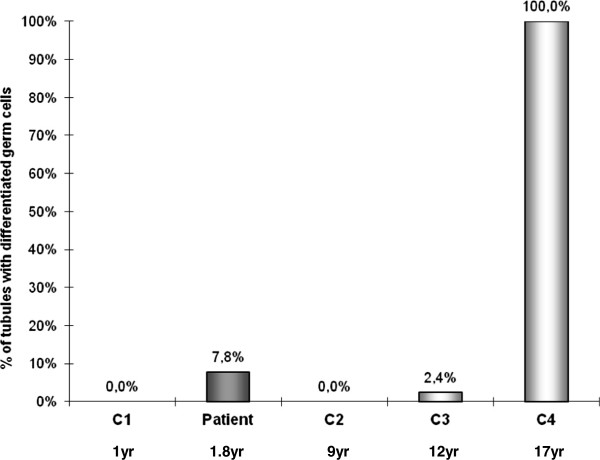
**Percentages of tubules with germ cell differentiation evaluated in seminiferous tubules of the Hurler syndrome patient and controls according to the age at the time of testicular biopsy.**

Spermatocytes I were detected in 7.8% of seminiferous cord sections in our HS patient (Figures 3 and 2D2). Meiotic germ cells represented 0.9% of intra-tubular cells. Germ cell differentiation up to the spermatocyte I stage was not observed in the seminiferous tubules of the youngest pre-pubertal controls (1 and 9-year-old controls respectively, Figures 3 and 2A2 and B2) and was only identified in 2.4% of tubules of the 12-year-old patient with germ cells at pachytene stage representing 0.2% of intra-tubular cells (Figures 3 and 2C2). For the 17-year-old patient, 67.5% of intra tubular cells were meiotic germ cells (Figure 2E2).

For the Hurler patient and the controls, AR was always expressed in Leydig and myoid cells. Furthermore, AR was not observed in Sertoli cells of 1-year (C1) and 9 (C2) -year-old controls) but was detected in the 12-year control. Our patient presented a major nuclear expression of AR (Figure 2A4, B4, C4 and E4). Moreover, AMH was strongly expressed in Sertoli cells of the youngest controls (C1, C2 and C3), weakly expressed in our Hurler patient and was undetectable in the oldest control (C4) (Figure 2A3, B3, C3, D3 and E3). However, most of Sertoli cell nuclei of our patient had an infantile morphological aspect with one or two small nucleoli and a rounded or ovoid shape. According to these criteria, Sertoli cell maturity of our Hurler patient was comparable to the 12-year-old control (C3). The expression of 3β-HSD was confined to the cytoplasm of the Leydig cells, was relatively intense in C2 and C3 controls and markedly expressed in pubertal C4 control but was not detectable in the youngest C1 control. Leydig cells of our Hurler patient expressed moderately 3β-HSD (Figure 2A5, B5, C5, D5 and E5).

## Discussion and conclusion

Our study explored, to the best of our knowledge and for the first time, the testicular development of a pre-pubertal patient with mucopolysaccharidosis that revealed a precocious initiation of spermatogenesis. Seminiferous tubules presented histological modifications observed normally in seminiferous tubules of boys during the initiation of the first waves of spermatogenesis. However, the plasmatic hormonal profile of our patient was not in agreement with the histological features of seminiferous tubules because the plasmatic level of inhibin B was elevated at the time of the biopsy but the plasmatic level of AMH remained moderately increased. After HSCT, FSH increased slightly while inhibin B and AMH decreased significantly (Table 1).

Between birth and the onset of puberty in humans, seminiferous tubules do not usually show major changes. Tubular diameter remains stable (~50-60 μm) [17]; Sertoli cells show typically immature features with oval and elongated nuclei associated to a regular shape [16] and a high level of AMH expression [18]; AR is not or slightly expressed in Sertoli cells [19]; furthermore, germ cell population, initially represented by gonocytes and subsequently by spermatogonia, proliferates by mitosis [20]. However, the pre-pubertal testis should not be considered as a quiescent organ because the testis exhibits numerous changes during infancy to prepare the pubertal maturation [21]. During the pubertal development, the enlargement of testicular size relies mainly on the increase of seminiferous tubule volume depending on the tubule diameter and the tubular length. Testis volume is directly dependant on germ cell proliferation and Sertoli cell number [22]. The tubular diameter observed in our patient corresponded to a tubular diameter of a testis during the pubertal development when compared with controls. It is well established that testicular volume closely correlates with the degree of testicular maturation.

Meiotic germ cells were detected in seminiferous tubules of our HS patient. Primary spermatocytes had yet been observed in seminiferous tubules of 4, 8 and 9-year-old pre-pubertal normal boys, but never in boys aged less than 2 years [23]. During the normal development of the testicle, a marked proliferation of spermatogonia occurs at 3-4 and 8-9 years [24–26], generally interpreted as premature failed attempts of spermatogenesis initiation. At about 4 and 9 years, a slight increase of FSH plasmatic level that might be involved in the initiation of the meiotic process has also been reported [27, 28]. This incomplete achievement of the spermatogenetic process may be related not solely to an insufficient production of testosterone [29] but also to an immature status of Sertoli cell. Our HS patient should be an example of such physiological process but these data would not explain the premature modification of AR and AMH expression in Sertoli cells.

At the onset of puberty, testosterone induces Sertoli cell morphological [30] and functional maturation with a significant decrease of AMH expression [31] and a highly expression of AR [19]. In our patient, 3β-HSD, a typical marker of Leydig cell precursors or mature Leydig cells, was moderately expressed in the interstitial compartment. These data confirmed the hypothesis of a probable discrete rise in intra-testicular testosterone due to a probable premature maturation of Leydig cell function, even if the testosterone blood level was not increased in our patient. This discrete amount of testosterone promotes the maturation of Sertoli cells, the initiation of spermatogenesis and the increase of seminiferous tubule diameter, as observed in our patient [21, 22]. During the physiological onset of puberty, intra-testicular testosterone level increases more precociously than its plasmatic level [22].

In addition, Sertoli cells of our patient maintained an infantile morphology, as for C3 control boy considered at the onset of puberty, [15] but a level of expression of AMH and AR close to mature Sertoli cells. Therefore, AMH was weakly expressed and AR was markedly detected. Moreover, inhibin B produced by Sertoli cells and known to be significantly increased at pubertal stage II compared with pre-pubertal period (stage I) [32] was measured at a high concentration in the plasma of our patient before HSCT. Our patient did not present any clinical signs of precocious puberty with no enlargement of testicular size but this parameter was indirectly assessed using manual palpation. In a few published reports, precocious puberty was diagnosed or suspected in 4 (15%) out of 27 young males and females with HS after HSCT. Two 7-year-old girls had clinical signs of precocious puberty and the two other patients, sex not mentioned, presented biochemical hormonal plasmatic profile consistent with a precocious puberty [10]. In addition, precocious puberty has been yet reported in five boys with type IIIA mucopolysaccharidosis. Precocious puberty was diagnosed between 5.9 to 9.6 years. Basal LH and testosterone plasmatic levels were always increased and basal FSH plasmatic level was elevated for four patients [8, 9].

A lesion of the central nervous system has been detected in 94% of boys with central precocious puberty (CPP) [11]. Hurler syndrome induced alterations of the central nervous system such as cystic areas in the centrum semiovale, peritrigonal white matter, corpus callosum or pericallosal region [33] and ventricular enlargement with communicating hydrocephalus in advanced stages [34]. The accumulation of glycosaminoglycans may cause CPP by interfering with neural pathways that inhibit Gonadotropin Releasing Hormon (GnRH) generation, by acting on the hypothalamus or the pituitary gland through an increased intracranial pressure [7] and by disturbing the activity of several cytokines involved in GnRH regulation [35, 36].

In our report, the basal LH, FSH and testosterone blood levels were not increased at the time of the biopsy and we did not have any complementary data from the follow-up showing an activation of the GnRH neuron in our patient. Therefore, it is difficult to conclude that glycosaminoglycans overproduction and central nervous system lesions may have activated the hypothalamic-pituitary-gonadal axis, responsible for the premature initiation of spermatogenesis. A local effect induced by the heparane sulphate accumulation is also possible. Indeed, the proteoglycans are present in the seminiferous tubule basement membrane [37] and in the membrane of Sertoli cells [38]. The function of the proteoglycans remains to be cleared up, but we know, for example, that spermatogonia and Sertoli cells require follistatin to modulate the actions of activin A during the development of the testicle [39] and that the active form of follistatin binds to the cell surfaces or the basement membranes with a strong affinity for proteoglycans [40].

Clinical and biochemical follow-up of our patient may contribute to determine if the abnormal histological feature observed in seminiferous tubules that did not correspond to the physiological age of our HS patient is predictive of CCP. CCP treatment is indicated to prevent progression of puberty. Thus, GnRHa therapy in two patients affected by MPS type IIIA had a beneficial effect on height but also on behavioural problems such as hyperactivity, destructive behaviour and sleep disturbances [9].

Testicular tissue banking of HS patients performed before HSCT, may allow fertility preservation but can also be useful for the detection of an abnormal gonad development and to plan hormonal therapy if necessary. However, this genetic disease remains exceptional. Our case report also confirms that Sertoli cell maturation observed at the initiation of spermatogenesis precedes the elevation of testosterone plasmatic level and the clinical signs of puberty.

## Consent

Written consent was obtained from the parents or legal guardians of the patients for publication of this Case report and any accompanying images.

## Abbreviations

AR: Androgen receptor
AMH: Anti-müllerian hormone
3β-HSD: 3β-hydroxysteroid dehydrogenase
CPP: Central precocious puberty
FSH: Follicle-stimulating hormone
GAG: Glycosaminoglycans
GnRH: Gonadotropin releasing hormon
HSCT: Haematopoietic stem cell transplantation
HES: Haematoxylin-eosin-saffron
LH: Luteinising hormone
MPS IH: Mucopolysaccharidose de type IH
RT: Room temperature.

## References

[1] Bach G, Friedman R, Weissmann B, Neufeld EF (1972). The defect in the Hurler and Scheie syndromes: deficiency of alpha-L-iduronidase. Proc Natl Acad Sci U S A.

[2] Moore D, Connock MJ, Wraith E, Lavery C (2008). The prevalence of and survival in mucopolysaccharidosis I: Hurler, Hurler-Scheie and Scheie syndromes in the UK. Orphanet J Rare Dis.

[3] Vellodi A, Young EP, Cooper A, Wraith JE, Winchester B, Meaney C, Ramaswami U, Will A (1997). Bone marrow transplantation for mucopolysaccharidosis type I: experience of two British centres. Arch Dis Child.

[4] Staba SL, Escolar ML, Poe M, Kim Y, Martin PL, Szabolcs P, Allison-Thacker J, Wood S, Wenger DA, Rubinstein P, Hopwood JJ, Krivit W, Kurtzberg J (2004). Cord-blood transplants from unrelated donors in patients with Hurler’s syndrome. N Engl J Med.

[5] Grewal SS, Wynn R, Abdenur JE, Burton BK, Gharib M, Haase C, Hayashi RJ, Shenoy S, Sillence D, Tiller GE, Dudek ME, van Royen-Kerkhof A, Wraith JE, Woodard P, Young GA, Wulffraat N, Whitley CB, Peters C (2005). Safety and efficacy of enzyme replacement therapy in combination with hematopoietic stem cell transplantation in Hurler syndrome. Genet Med.

[6] Cox-Brinkman J, Boelens JJ, Wraith JE, O’meara A, Veys P, Wijburg FA, Wulffraat N, Wynn RF (2006). Haematopoietic cell transplantation (HCT) in combination with enzyme replacement therapy (ERT) in patients with Hurler syndrome. Bone Marrow Transplant.

[7] Kaplan SL, Grumbach MM (1990). Clinical review 14: pathophysiology and treatment of sexual precocity. J Clin Endocrinol Metab.

[8] Tylki-Szymańska A, Metera M (1995). Precocious puberty in three boys with Sanfilippo A (mucopolysaccharidosis III A). J Pediatr Endocrinol Metab.

[9] Concolino D, Muzzi G, Pisaturo L, Piccirillo A, Di Natale P, Strisciuglio P (2008). Precocious puberty in Sanfilippo IIIA disease: diagnosis and follow-up of two new cases. Eur J Med Genet.

[10] Polgreen LE, Tolar J, Plog M, Himes JH, Orchard PJ, Whitley CB, Miller BS, Petryk A (2008). Growth and endocrine function in patients with Hurler syndrome after hematopoietic stem cell transplantation. Bone Marrow Transplant.

[11] Pescovitz OH, Comite F, Hench K, Barnes K, McNemar A, Foster C, Kenigsberg D, Loriaux DL, Cutler GB (1986). The NIH experience with precocious puberty: diagnostic subgroups and response to short-term luteinizing hormone releasing hormone analogue therapy. J Pediatr.

[12] Marshall WA, Tanner JM (1970). Variations in the pattern of pubertal changes in boys. Arch Dis Child.

[13] Milazzo JP, Vaudreuil L, Cauliez B, Gruel E, Massé L, Mousset-Siméon N, Macé B, Rives N (2008). Comparison of conditions for cryopreservation of testicular tissue from immature mice. Hum Reprod.

[14] Milazzo JP, Travers A, Bironneau A, Safsaf A, Arnoult C, Macé B, Boyer O, Rives N (2010). Rapid screening of cryopreservation protocols for murine prepubertal testicular tissue by histology and PCNA immunostaining. J Androl.

[15] Nistal M, De Mora JC, Paniagua R (1998). Classification of several types of maturational arrest of spermatogonia according to Sertoli cell morphology: an approach to aetiology. Int J Androl.

[16] Rives N, Milazzo JP, Perdrix A, Castanet M, Joly-Hélas G, Sibert L, Bironneau A, Way A, Macé B (2013). The feasibility of fertility preservation in adolescents with Klinefelter syndrome. Hum Reprod.

[17] Müller J, Skakkebaek NE (1983). Quantification of germ cells and seminiferous tubules by stereological examination of testicles from 50 boys who suffered from sudden death. Int J Androl.

[18] Rajpert-de-Meyts E, Jorgensen N, Graem N, Müller J, Cate RL, Skakkebaek NE (1999). Expression of anti-müllerian hormone during normal and pathological gonadal development: association with differentiation of sertoli and granulosa cells. J Clin Endocrinol Metab.

[19] Rey RA, Musse M, Venara M, Chemes HE (2009). Ontogeny of the androgen receptor expression in the fetal and postnatal testis: its relevance on Sertoli cell maturation and the onset of adult spermatogenesis. Microsc Res Tech.

[20] Sharpe M, McKinnel C, Kilvin C, Fisher S (2003). Proliferation and funtional maturation of sertoli cells, and their relevance to disorders of testis function in adulthood. Reproduction.

[21] Chemes HE (2001). Infancy is not a quiescent period of testicular development. Int J Androl.

[22] Rey R (1999). The prepubertal testis: a quiescent or a silently active organ?. Histol Histopathol.

[23] Nistal M, Paniagua R (1984). Occurrence of primary spermatocytes in the infant and child testis. Andrologia.

[24] Mancini RE, Narbaitz R, Lavieri JC (1960). Origin and development of the germinative epithelium and sertoli cells in the human testis: cytological, cytochemical, and quantitative study. Anat Rec.

[25] Vilar O, Rosemberg E, Paulsen CA (1970). Histology of the human testis from neonatal period to adolescence. In advances in experimental medicine and biology. The Human Testis, Volume 10.

[26] Hedinger E (1982). Histopathology of undescended testes. Eur J Pediatr.

[27] Fairman C, Winter JSD, Grumbach MM, Grave GD, Mayer FE (1974). Gonadotropins and sex hormone patterns in puberty. The Control of the Onset of Puberty.

[28] Kulin HE, Santner SJ (1977). Timed urinary gonadotropin measurements in normal infants, children, and adults, and in patients with disorders of sexual maturation. J Pediatr.

[29] Swerdloff RS, Heber D, Burger H, de Kretser D (1981). Endocrine control of testicular function from birth to puberty. Comprehensive Endocrinology: the testis.

[30] Chemes HE, Dym M, Raj HG (1979). Hormonal regulation of sertoli cell differentiation. Biol Reprod.

[31] Rey R, Lordereau-Richard I, Carel JC, Barbet P, Cate RL, Roger M, Chaussain JL, Josso N (1993). Anti-müllerian hormone and testosterone serum levels are inversely during normal and precocious pubertal development. Clin Endocrinol Metab.

[32] Andersson AM, Juul A, Petersen JH, Müller J, Groome NP, Skakkebaek NE (1997). Serum inhibin B in healthy pubertal and adolescent boys: relation to age, stage of puberty, and follicle-stimulating hormone, luteinizing hormone, testosterone, and estradiol levels. J Clin Endocrinol Metab.

[33] Afifi AK, Sato Y, Waziri MH, Bell WE (1990). Computed tomography and magnetic resonance imaging of the brain in Hurler’s disease. J Child Neurol.

[34] Panteliadis CP, Karatza ED, Tzitiridou MK, Koliouskas DE, Spiroglou KS (2003). Lissencephaly and mongolian spots in Hurler syndrome. Pediatr Neurol.

[35] Kreuger J, Salmivirta M, Sturiale L, Giménez-Gallego G, Lindahl U (2001). Sequence analysis of heparan sulfate epitopes with graded affinities for fibroblast growth factors 1 and 2. J Biol Chem.

[36] González-Martínez D, Kim SH, Hu Y, Guimond S, Schofield J, Winyard P, Vannelli GB, Turnbull J, Bouloux PM (2004). Anosmin-1 modulates fibroblast growth factor receptor 1 signaling in human gonadotropin-releasing hormone olfactory neuroblasts through a heparan sulfate-dependent mechanism. J Neurosci.

[37] Bichoualne L, Thiébot B, Langris M, Barbey P, Oulhaj H, Bocquet J (1994). Membrane associated proteoglycans in rat testicular peritubular cells. Mol Cell Biochem.

[38] Brucato S, Fagnen G, Villers C, Bonnamy PJ, Langris M, Bocquet J (2001). Biochemical characterization of integral membrane heparan sulfate proteoglycans in sertoli cells from immature rat testis. Biochim Biophys Acta.

[39] Nakamura T, Takio K, Eto Y, Shibai H, Titani K, Sugino H (1990). Activin-binding protein from rat ovary is follistatin. Science.

[40] Esch FS, Shimasaki S, Mercado M, Cooksey K, Ling N, Ying S, Ueno N, Guillemin R (1987). Structural characterization of follistatin: a novel follicle-stimulating hormone release-inhibiting polypeptide from the gonad. Mol Endocrinol.

